# Puerarin Targets HIF-1α to Modulate Hypoxia-Related Sphingolipid Metabolism in Diabetic Hepatopathy via the SPTLC2/Ceramide Pathway

**DOI:** 10.3390/ph18030398

**Published:** 2025-03-12

**Authors:** Mangui Cai, Wenxi Lai, Huien Chen, Dongmin Cao, Boyan Zhang, Feng Wang, Minghua Xian, Shumei Wang

**Affiliations:** 1 School of Chinese Materia Medica, Guangdong Pharmaceutical University, Guangzhou 510006, China; 2 Key Laboratory of Digital Quality Evaluation of Chinese Materia Medica of State Administration of TCM, School of Chinese Materia Medica, Guangdong Pharmaceutical University, Guangzhou 510006, China; 3 Engineering & Technology Research Center for Chinese Materia Medica Quality of the Universities of Guangdong Province, School of Chinese Materia Medica, Guangdong Pharmaceutical University, Guangzhou 510006, China; 4 Traditional Chinese Medicine Resource Germplasm Bank Management Center, Yunfu 527300, China

**Keywords:** Puerarin, diabetic hepatopathy, HIF-1α, metabolic pathways, ceramide

## Abstract

**Background and Objectives**: Diabetic hepatopathy, characterized by hepatic hypoxia and metabolic dysregulation, has a rising global incidence and prevalence, with limited effective treatments. Hepatic hypoxia activates hypoxia-inducible factor-1 alpha (HIF-1α), regulating sphingolipid metabolism and elevating ceramide, a key factor in insulin resistance. Puerarin (Pue), a flavonoid derived from Pueraria lobata, exhibits therapeutic effects in diabetes, but its effects on hypoxia-related hepatic metabolism are unclear. This study investigates Pue’s mechanisms in modulating hepatic metabolism, focusing on HIF-1α and sphingolipid metabolism. **Methods**: Using bioinformatics and molecular docking, HIF-1α was identified as a key target in diabetic liver disease, confirmed via drug affinity responsive target stability. In vitro experiments utilized insulin-resistant HepG2 cells to assess glucose intake and HIF-1α expression. In vivo, type 2 diabetes mellitus (T2DM) was induced in mice using a high-fat diet and streptozotocin injections. Pue administration was evaluated for its effects on fasting blood glucose, oral glucose tolerance, and hepatoprotective effects. Liver metabolomics and qPCR/Western blot analyses were conducted to assess metabolic pathways. **Results**: Pue increased glucose uptake in HepG2 cells and bound HIF-1α. Pue reduced HIF-1α expression in HepG2 cells, an effect attenuated by the HIF-1α stabilizer DMOG. Pue improved fasting blood glucose, oral glucose tolerance, and hepatoprotective effects in T2DM mice, which DMOG reversed. Metabolomics revealed that Pue modulates sphingolipid metabolism, decreasing ceramide content. qPCR and Western blot results confirmed that Pue dramatically decreases HIF-1α and SPTLC2 expression. **Conclusions**: Pue improves diabetic hepatopathy by reducing ceramide expression through the HIF-1α/SPTLC2 pathway, offering a novel therapeutic strategy for diabetes management.

## 1. Introduction

Diabetes mellitus and its complications are gradually becoming a significant global health concern [1]. Among all types of diabetes, type 2 diabetes mellitus (T2DM) has the highest prevalence, accounting for approximately 90% of diabetic incidence [2]. Diabetic hepatopathy is one of the main complications of T2DM. The Japan Society of Diabetes Mellitus reported that 13.3% of deaths among patients with diabetes were attributable to liver disease [3]. At present, there is no specific drug to treat diabetic hepatopathy. Clinically, patients with this disease mainly regulate metabolic disorders by improving lifestyles (diet, exercise, etc.). Therefore, it is necessary to find a drug to treat diabetic hepatopathy [4]. The liver is one of the most important organs for maintaining the body’s glycolipid metabolism and is usually the first to develop insulin resistance (IR) in diabetes. Excessive nutrient (high fat and sugar) intake results in increased hepatocyte lipid deposition, which leads to hepatocyte swelling and reduces volume within the hepatic sinusoids, reducing oxygen supply to the hepatocytes [5,6,7]. This physiological process leads to a hypoxic environment for liver cells, resulting in increased levels of reactive oxygen species (ROS) in liver cells [8]. ROS is a key process leading to liver injury, which causes liver fibrosis by destroying cellular lipids, proteins, and DNA and leads to hepatocyte necrosis and apoptosis [9].

Our studies have highlighted the importance of hub genes in the pathophysiology of diabetic hepatopathy, with hypoxia-inducible factor-1α (HIF-1α) and C-X-C motif chemokine 2 (CXCL2) emerging as key players. HIF-1α, in particular, has been implicated in regulating glucose metabolism and is a potential therapeutic target. HIF-1α, as a key transcription factor, is involved in regulating multiple biological processes associated with diabetes, including but not limited to glucose metabolism, lipid metabolism, angiogenesis, apoptosis, and inflammatory responses [10,11]. HIF-1α affects liver disease by regulating genes involved in glucose and lipid metabolism. Under the condition of liver hypoxia, HIF-1α may regulate liver gene coding, thereby affecting liver glucose transport and fructose production and further affecting the development of liver disease [12]. HIF-1α is key in the relationship between lipid metabolism and IR. HIF-1α is a major regulator of the adaptive response to hypoxia and is ubiquitinated by the ubiquitin ligase complex and subjected to proteasomal degradation in a normal environment. Because it requires molecular oxygen for its degradation mechanism, it can remain stable in hypoxic environments. It binds to HIF-1β to form a heterodimer and functions as a transcription factor. This can stimulate the transcription of genes related to glucose transport, glycolysis, ceramide synthetase, and other genes, thus influencing the disease. A high-fat diet has led to increased HIF-1α expression in the liver [13]. Hepatocytes cultured in a hypoxic environment (O_2_ less than 1%) showed steatosis [14]. Therefore, correcting liver hypoxia through drug intervention provides new ideas and methods for treating diabetic hepatopathy.

**Figure 1:**
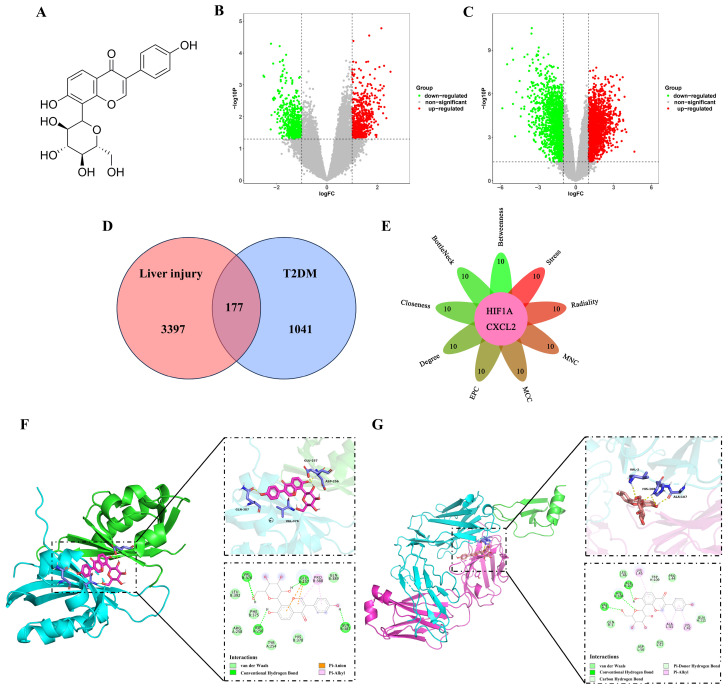
Hub gene screening for liver injury in T2DM and molecular docking of Puerarin with hub genes. (**A**) The chemical structure of Pue; (**B**) volcano plot of DEGs in GSE23343; (**C**) volcano plot of DEGs in GSE54255; (**D**) Venn diagram of DEGs between T2DM with liver injury; (**E**) hub genes obtained from nine algorithms in CytoHubba; (**F**) molecular docking of Pue and HIF-1α; (**G**) molecular docking analysis of Pue and CXCL2.

*Pueraria lobata* (Willd.) Ohwi is a traditional Chinese medicine with ameliorative effects on diabetes [15]. Puerarin (Pue), an isoflavone component, has attracted much attention and research because of its high content and activity in Pueraria lobata [16,17]. The chemical structure of Pue is shown in Figure 1A. Studies have shown that Pue not only improves fasting blood glucose and glucose tolerance in diabetic mice but also has protective effects on pancreatic β-cells. These protective effects may be achieved through various mechanisms such as activation of the PI3K/AKT signaling pathway, enhancement of the GLP-1 signaling pathway, attenuation of oxidative stress damage, and inhibition of β-cell apoptosis [18,19,20]. Pueraria lobata starch and Pueraria flavonoids alleviate high-fat high-cholesterol diet-induced non-alcoholic fatty liver disease in mice [21,22]. Despite numerous findings, the mechanism of action of Pue, especially its effect on HIF-1α under hypoxic conditions in the liver caused by diabetic hepatopathy, remains unclear. Therefore, an in-depth study of the mechanisms of how Pue affects HIF-1α may reveal new therapeutic targets and provide new strategies for the treatment of diabetes and its complications.

In this study, we employed an integrated approach combining bioinformatics, molecular docking, and metabolomics to investigate the effects and mechanisms of Pue in diabetic hepatopathy. We speculated that HIF-1α may be a key target in diabetic hepatopathy through bioinformatics analysis. Molecular docking studies were performed to explore the binding interactions between Pue and HIF-1α. In vitro experiments were conducted to assess the effect of Pue on glucose intake in insulin-resistant HepG2 cells, and the binding affinity of Pue for HIF-1α was confirmed by drug affinity responsive target stability (DARTS) and cellular immunofluorescence assays. Using an in vivo diabetes model, we observed the effects of Pue on fasting blood glucose levels, oral glucose tolerance, and liver function. We also examined whether these effects were reversed by the HIF-1α stabilizer Dimethyloxallyl Glycine (DMOG). Finally, we utilized untargeted metabolomics to uncover the mechanisms underlying the therapeutic effects of Pue.

## 2. Results

### 2.1. Analysis of the Hub Genes Between T2DM and Liver Injury Using Bioinformatics

Based on the differential expression criteria, a total of 1218 differentially expressed genes (DEGs) were identified from the GSE23343 dataset, consisting of 629 upregulated genes and 589 downregulated genes (Figure 1B). From the GSE54255 dataset, 3574 DEGs were identified, including 1964 upregulated genes and 1610 downregulated genes (Figure 1C). Then, a total of 177 DEGs were identified in the intersection of the two datasets (Figure 1D). Nine algorithms (Betweenness, BottleNeck, Closeness, Degree, EPC, MCC, MNC, Radiality, and Stress) were utilized to identify hub genes by CytoHubba. As a result, two hub genes, HIF-1α and CXCL2, were identified, providing key insights into the functional interactions within the network (Figure 1E). Moreover, the results of molecular docking studies provided evidence of a strong binding interaction between Pue and HIF-1α, with the binding energy below −5 kcal/mol (Figure 1F). This interaction is characterized by specific hydrogen bonding in key amino acid residues: ASP256, GLU257, VAL376, and GLN387. In contrast, the molecular docking results for Pue and CXCL2 revealed a binding energy greater than −5 kcal/mol (Figure 1G).

### 2.2. Puerarin Increases Glucose Intake in Insulin-Resistant Hepatocytes HepG2

**Figure 2:**
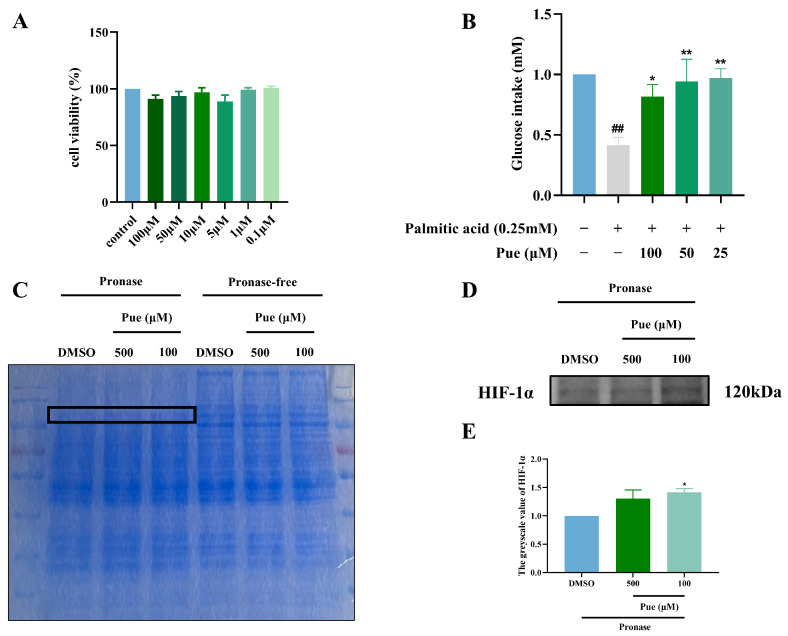
Puerarin enhances glucose uptake in insulin-resistant HepG2 cells and demonstrates binding affinity for HIF-1α. (**A**) Toxicity test of Pue on HepG2 cell; (**B**) Pue ameliorating insulin resistance in IR-HepG2 cell; (**C**) identification of Pue target proteins using DARTS (The black box: Coomassie brilliant blue stripes with differences); (**D**) validation of the binding of Pue and HIF-1α; (**E**) greyscale value of HIF-1α. Data are presented as means ± SEM (n = 3). ^##^ *p* < 0.01, versus the control group. * *p* < 0.05, ** *p* < 0.01, versus the model group.

Pue had no significant effect on the viability of HepG2 cells in the concentration range of 0.1 μM–100 μM (Figure 2A). The glucose intake of HepG2 cells in the model group significantly decreased by 58.46% (*p* < 0.01) compared to the control group, indicating the successful establishment of the insulin-resistant HepG2 (IR-HepG2) cell model. Additionally, the glucose intake of IR-HepG2 cells was significantly higher in various Pue dosage groups compared to the model group, suggesting that Pue has the potential to improve the insulin resistance of HepG2 cells (Figure 2B).

### 2.3. Puerarin Exhibits a Binding Affinity for HIF-1α

According to the results of DARTS, the Pue group showed a significant difference, shown in the black box (Figure 2C). Furthermore, DARTS-WB analysis showed that Pue may interact with HIF-1α (Figure 2D,E).

### 2.4. Inhibition of HIF-1α by Puerarin Ameliorates Insulin Resistance in HepG2 Cells

**Figure 3:**
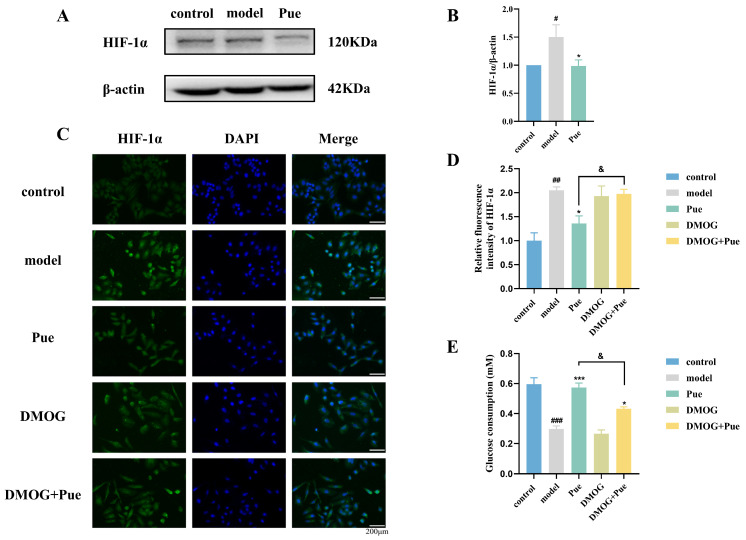
Inhibition of HIF-1α by Puerarin ameliorates insulin resistance in HepG2 cells. (**A**,**B**) Pue inhibition of the expression of HIF-1α protein in IR-HepG2 cell; (**C**,**D**) fluorescence expression of HIF-1α in IR-HepG2 cell; (**E**) DMOG reversing the improvement of IR-HepG2 glucose depletion by Pue. The concentration of Pue was 100 μM. Data are presented as means ± SEM (n = 3–4). ^#^ *p* < 0.05, ^##^ *p* < 0.01, ^###^ *p* < 0.001, versus the control group. * *p* < 0.05, *** *p* < 0.001, versus the model group. ^&^ *p* < 0.05, versus the Pue group.

In the IR-HepG2 cell model, the expression of the HIF-1α protein was significantly increased. However, after exposure to Pue, the expression of the HIF-1α protein was significantly decreased (Figure 3A,B). Cellular immunofluorescence assays further corroborated these findings and showed a significant increase in HIF-1α expression in the model group, but a decrease in HIF-1α expression after Pue exposure. This effect was reversed when DMOG was given (Figure 3C,D). This suggests that Pue has the ability to inhibit HIF-1α protein expression in IR-HepG2 cells. In addition, glucose intake experiments showed that the effect of Pue on improving glucose intake in IR-HepG2 was reversed after DMOG treatment (Figure 3E). The above results reveal that Pue can improve insulin resistance in HepG2 cells by inhibiting HIF-1α.

### 2.5. Effect of Puerarin on Fasting Blood Glucose and Oral Glucose Tolerance in Diabetic Mice, Reversed by HIF-1α Stabilizer DMOG

**Figure 4:**
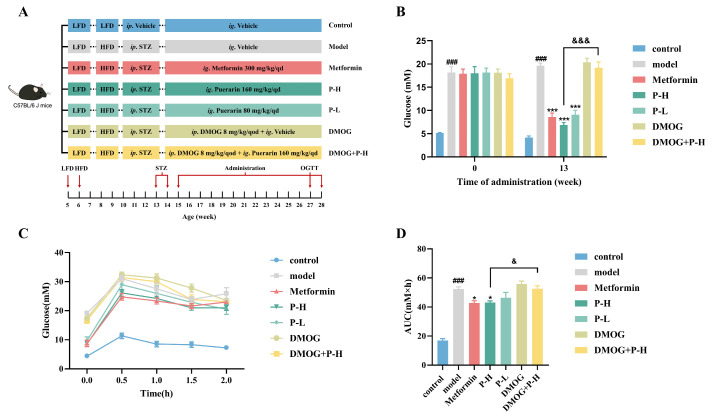
Effect of Puerarin on fasting blood glucose and oral glucose tolerance in diabetic mice, reversed by HIF-1α stabilizer DMOG. (**A**) Groups and schematic diagram of the animal experiment; (**B**) fasting blood glucose levels at 0 and 13th weeks; (**C**) variation in OGTT in 2 h at 12th week; (**D**) statistical area under the OGTT curve. Data are presented as means ± SEM (n = 8). ^###^ *p* < 0.001, versus the control group. * *p* < 0.05, *** *p* < 0.001, versus the model group. ^&^ *p* < 0.05, ^&&&^ *p* < 0.001, versus the P-H group.

The above in vitro experiments showed that Pue could inhibit HIF-1α to improve insulin resistance in HepG2 cells. Subsequently, we verified whether the inhibition of HIF-1α by Pue improved liver injury in T2DM mice. Streptozotocin (STZ) is widely used as a diabetic causative agent because it can damage islet β cells by triggering the immune system response [23]. Therefore, the mouse model of T2DM was established with a high-fat diet combined with STZ injection [24]. The animal experimental dosing schedule is depicted in Figure 4A. Following the administration of STZ, the blood glucose among mice in the experimental group was significantly increased by 265.31% compared to the control group (5.16 ± 0.10 mM) (*p* < 0.05), confirming the successful establishment of the diabetic mouse model. Treatment with Pue and metformin led to a marked decrease in blood glucose levels in diabetic mice compared to the control group, with the hypoglycemic effect of Pue being reversed by DMOG (Figure 4B). Furthermore, in the oral glucose tolerance assessment, initial blood glucose levels at 0 h were significantly reduced in all Pue-treated and metformin groups compared to the model group. At 0.5 h, the blood glucose of mice in all groups increased sharply. For the model, DMOG, and DMOG + P-H groups, glucose levels exceeded 30 mM. Notably, the P-H group (26.10 ± 1.17 mM) exhibited a markedly reduced blood glucose concentration compared to the DMOG + P-H group (31.41 ± 1.30 mM) (*p* < 0.05). At 2 h, Pue treatment across all dosages resulted in a pronounced decrease in blood glucose levels when contrasted with the model group (25.85 ± 2.05 mM) (*p* < 0.05). The P-H group (21.01 ± 0.90 mM) particularly stood out with an even lower glucose level than the DMOG + P-H group (23.11 ± 0.68 mM) (*p* < 0.05), as depicted in Figure 4C. The oral glucose tolerance test revealed a significant difference in the area under the curve (AUC) between the Pue dosage groups and the Metformin group (42.78 ± 1.56 mM × h) compared to the model group (52.45 ±1.43 mM × h) (*p* < 0.05). Additionally, there was a significant AUC difference between the DMOG + P-H group (52.57 ± 1.98 mM × h) and the P-H group (43.04 ± 1.12 mM × h) (*p* < 0.05) (see Figure 4D). These results indicate that Pue improves oral glucose tolerance in diabetic mice and DMOG reverses its effect.

### 2.6. The Hepatoprotective Effects of Puerarin in Diabetic Mice Are Reversed by the HIF-1α Stabilizer DMOG

**Figure 5:**
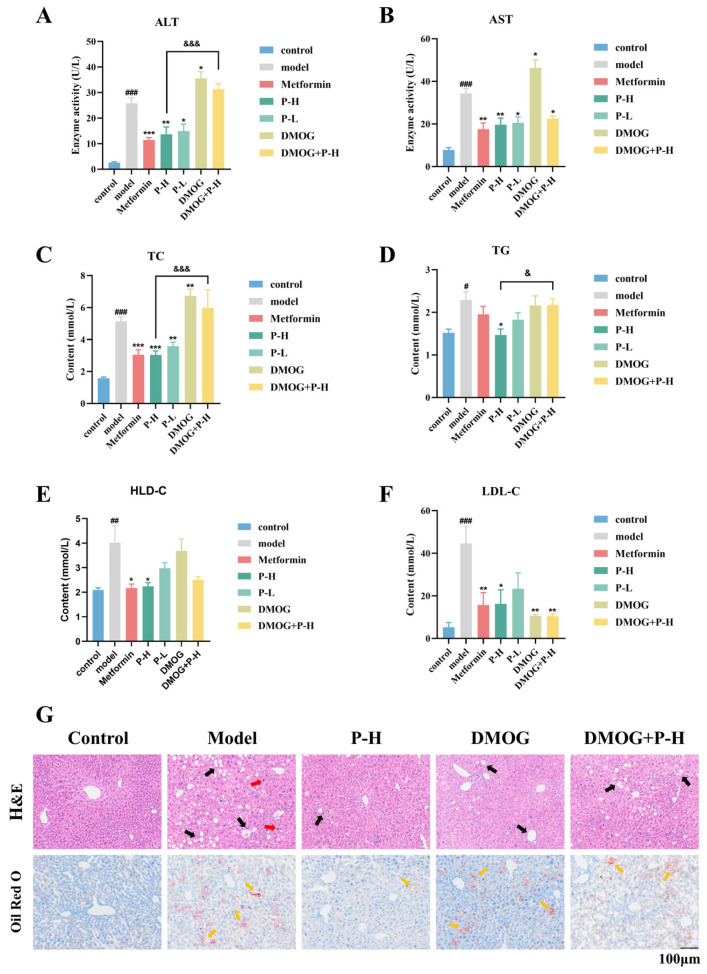
The hepatoprotective effects of Puerarin in diabetic mice are reversed by the HIF-1α stabilizer DMOG. (**A**) Plasma ALT levels in mice; (**B**) plasma AST levels in mice; (**C**) plasma TC levels in mice; (**D**) plasma TG levels in mice; (**E**) plasma HDL-C levels in mice; (**F**) plasma LDL-C levels in mice; (**G**) H&E staining and oil red O staining of mouse liver tissue. Black arrows indicate interstitial lipid vacuoles. Red arrows indicate lymphocytic infiltration. Yellow arrows indicate lipid droplet deposition. Data are presented as means ± SEM (n = 3 or 6). ^#^ *p* < 0.05, ^##^ *p* < 0.01, ^###^ *p* < 0.001, versus the control group. * *p* < 0.05, ** *p* < 0.01, *** *p* < 0.001, versus the Model group. ^&^ *p* < 0.05, ^&&&^ *p* < 0.001, versus the P-H group.

Aspartate aminotransferase (AST) and alanine aminotransferase (ALT) are the most commonly used indicators for evaluating liver function and liver injury [25]. The results showed that Pue significantly reduced ALT and AST levels in diabetic mice. While the ALT levels in the DMOG + P-H group (31.31 ± 2.18 U/L) were significantly higher than those in the P-H group (13.63 ± 2.92 U/L), there was no significant difference in AST (Figure 5A,B). Triglyceride (TG), total cholesterol (TC), low-density lipoprotein cholesterol (LDL-C), and high-density lipoprotein cholesterol (HDL-C) are important indicators for assessing blood lipid profiles [26]. Excessive accumulation of these lipids in the blood can lead to liver dysfunction and related metabolic disorders [27]. The results showed that Pue treatment led to a significant decrease in lipid levels in diabetic mice when compared to the model group. Compared to the P-H group (TG: 1.47 ± 0.14 mmol/L; TC: 3.04 ± 0.25 mmol/L), the DMOG + P-H group (TG: 2.18 ± 0.15 mmol/L; TC: 6.00 ± 0.45 mmol/L) showed significantly higher TG and TC levels, but no significant difference in LDL-C and HDL-C levels (Figure 5C–F). Pue administration reduced lymphocytic infiltration and interstitial lipid vacuole formation in the livers of diabetic mice, which was a marked improvement over the model group. However, DMOG administration resulted in persistent lymphocytic infiltration and lipid vacuoles in the livers of the DMOG and DMOG + P-H groups, with severity exceeding that of the P-H group. The results of oil red O staining indicated that Pue had beneficial effects, as it led to a significant reduction in lipid droplet deposition in the livers of P-H mice compared to the model group. In contrast, after the administration of DMOG, lipid droplet deposition in the livers of the DMOG and DMOG + P-H groups was significantly increased compared with that of the P-H group (Figure 5G). These results suggest that DMOG can counteract the effects of Pue in ameliorating diabetic liver injury and regulating blood lipids.

### 2.7. Metabolic Profiling Reveals Key Metabolic Pathways in the Treatment of Diabetic Mice with Puerarin

**Figure 6:**
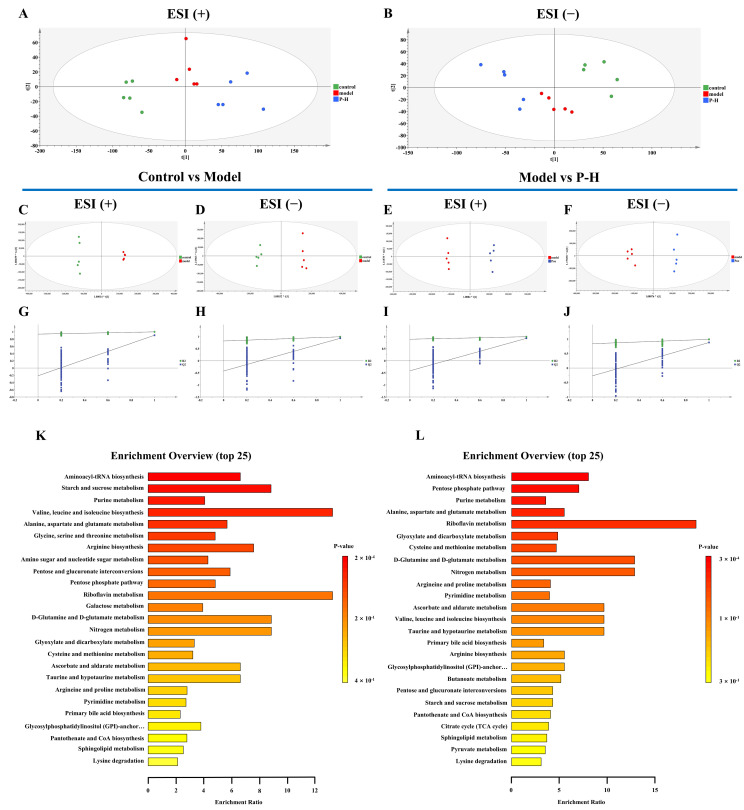
Metabolic profiling reveals key metabolic pathways in the treatment of diabetic mice with Puerarin. (**A**) PCA in positive ion mode; (**B**) PCA in negative ion mode; (**C**,**D**) OPLS-DA score plots for the control and model groups, where (**C**) is the positive ion mode and (**D**) is the negative ion mode; (**E**,**F**) OPLS-DA score plots for the model and P-H groups, where (**E**) is the positive ion mode and (**F**) is the negative ion mode; (**G**,**H**) permutation test of OPLS-DA for the control and model groups, where (**G**) is the positive ion mode and (**H**) is the negative ion mode; (**I**,**J**) permutation test of OPLS-DA for the model and P-H groups, where (**I**) is the positive ion mode and (**J**) is the negative ion mode; (**K**) pathway enrichment analysis of differential metabolites between the control and model groups; (**L**) pathway enrichment analysis of differential metabolites between the model and P-H groups. Data are presented as means ± SEM (n = 5).

To further explore the mechanism by which Pue exerts its therapeutic effects in diabetic mice, we used untargeted metabolomics. Principal component analysis (PCA) of hepatic tissue from the control, model, and Pue-treated (P-H) group demonstrated clear discrimination between groups in both positive and negative ion modes (Figure 6A,B). Orthogonal partial least squares discriminant analysis (OPLS-DA) further confirmed the metabolic divergence, with distinct clustering observed between control and model groups, as well as between model and P-H groups, highlighting the significant metabolic disruptions in diabetic mice and the restorative effects of Pue on these profiles (Figure 6C–F). The permutation test validation, with Q2 and the vertical coordinate intercept below 0, indicates that the model was not overfitted (Figure 6G–J). After differential metabolite screening and pathway enrichment analysis, a total of 25 related metabolic pathways were enriched in the control group and the model group and the model group and P-H group, respectively. Notably, sphingolipid metabolism and aminoacyl tRNA biosynthesis, purine metabolism, arginine biosynthesis, and cysteine and methionine metabolism emerged as key pathways that Pue significantly modulated (Figure 6K,L).

### 2.8. Puerarin Inhibits Ceramide Production Through the HIF-1α/SPTLC2 Pathway

**Figure 7:**
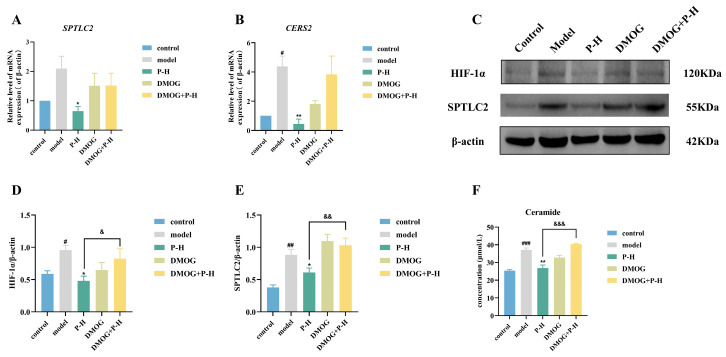
Puerarin inhibits ceramide production through the HIF-1α/SPTLC2 pathway. (**A**) The mRNA level of *SPTLC2* in liver tissue; (**B**) the mRNA level of *CERS2* in liver tissue; (**C**–**E**) the protein level of HIF-1α/SPTLC2 in liver tissue, identified by the Western blot. (**F**) The content of ceramide in liver tissue. Data are presented as means ± SEM (n = 3). ^#^ *p* < 0.05, ^##^ *p* < 0.01, ^###^ *p* < 0.001, versus the control group. * *p* < 0.05, ** *p* < 0.01, versus the model group. ^&^ *p* < 0.05, ^&&^ *p* < 0.01, ^&&&^ *p* < 0.001, versus the P-H group.

After a literature search of the above enriched related pathways, it was found that sphingolipid metabolism is one of the metabolic pathways closely related to diabetes, and its central hub is ceramide [28]. Serine palmitoyltransferase 2 (SPTLC2) and ceramide synthase 2 (CERS2) are two important enzymes in the sphingolipid metabolism pathway [29,30]. There was a tendency for an increase in the levels of *SPTLC2* mRNA and a significant increase in the levels of *CERS2* mRNA in the livers of mice in the model group. Both levels were significantly decreased after administration of Pue, which was reversed when DMOG was given (Figure 7A,B). Western blot results showed that HIF-1α and SPTLC2 protein expression was significantly increased in the liver tissues of mice in the model group compared to the control group. Liver tissue HIF-1α and SPTLC2 protein expressions were significantly decreased in mice in the P-H group compared to the model group. The reduction in HIF-1α and SPTLC2 protein expression by Pue was reversed when DMOG was administered (Figure 7C–E). Meanwhile, the ceramide content in the liver tissue of mice in the P-H group (26.89 ± 1.73 μmol/L) was significantly reduced compared with the model group (37.15 ± 1.05 μmol/L) (*p* < 0.01). When DMOG was administered, the effect of Pue in reducing ceramide was inhibited (Figure 7F). The results suggest that Pue inhibits ceramide production through the HIF-1α/SPTLC2 pathway.

## 3. Discussion

**Figure 8:**
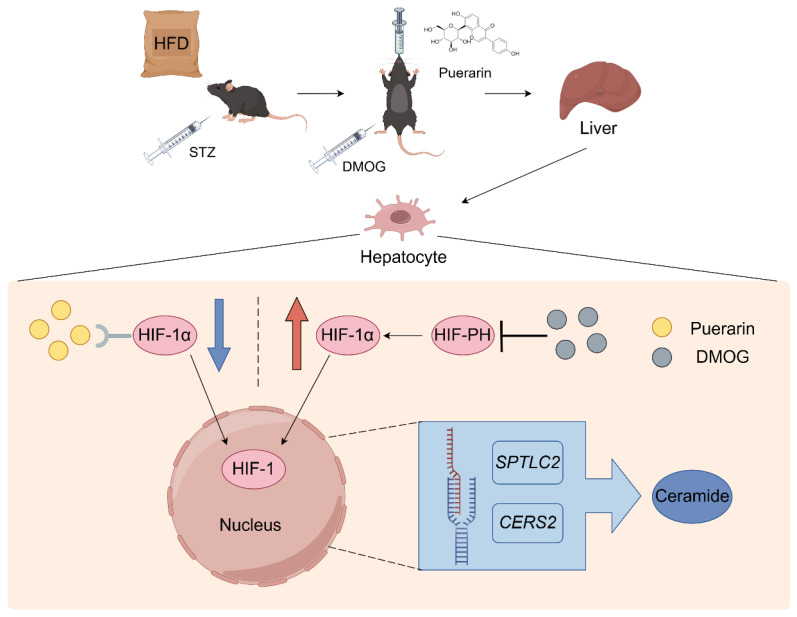
The mechanism diagram of how Puerarin targets HIF-1α to modulate SPTLC2/ceramide pathway (By Figdraw).

In this study, we have employed a comprehensive approach that integrates bioinformatics, molecular docking, and metabolomics to elucidate the therapeutic mechanisms of Pue in ameliorating IR and diabetes mellitus in mice, focusing on how Pue targets the HIF-1α/SPTLC2 pathway to modulate ceramide production (Figure 8).

Through bioinformatics analysis, we found that the hub genes of diabetic hepatopathy are HIF-1α and CXCL2. Molecular docking studies revealed a strong binding interaction between Pue and HIF-1α, suggesting a direct interaction that may underlie Pue’s therapeutic effects. In contrast, the binding energy between Pue and CXCL2 was greater than −5 kcal/mol, indicating a weaker interaction. These results directed our focus towards HIF-1α as the primary target of Pue in our subsequent in vitro and in vivo studies. The DARTS technique, commonly utilized for drug target screening [31], substantiated our molecular docking findings by demonstrating retention of the HIF-1α protein in the Pue group, indicative of a possible interaction between Pue and HIF-1α. HIF-1α, as a major regulator of the adaptive response to hypoxia, is one of the important transcription factors involved in IR and hepatic steatosis. Related studies have shown that a high-fat diet increases HIF-1α expression in the liver [13], which can induce hepatic steatosis [32,33]. Furthermore, the inhibition of HIF-1α prevents or reverses obesity-induced inflammation and IR [34]. This highlights the central role of HIF-1α in the pathological process of diabetes. Our study also found significantly elevated hepatic HIF-1α protein expression in diabetic mice, a result that is consistent with previous studies. When Pue was administered, its HIF-1α expression was reduced. We further validated the HIF-1α action of Pue using DMOG, which is an agent that leads to the accumulation and stabilization of the HIF-1 protein. The results of our in vivo experiments showed that the administration of DMOG inhibited the effects of Pue in lowering blood glucose and oral glucose tolerance in diabetic mice and reversed the effects of Pue in ameliorating hepatic tissue damage and hepatic lipid deposition.

In order to further study the mechanism of Pue in improving diabetic hepatopathy, we used untargeted metabolomics technology. The results of metabolomics enrichment analysis showed that sphingolipid metabolism was a related metabolic pathway between the model group and the Pue group. Sphingolipids mainly include sphingolipids, ceramides, and gangliosides, among which ceramides are the central hub of sphingolipid metabolism [28]. Ceramides are key players in lipotoxicity [35], and their accumulation in obese or dyslipidemic patients triggers tissue dysfunction. It has been reported that plasma and tissue ceramide concentrations are strongly associated with the risk of T2DM, hepatic steatosis, and cardiovascular disease [36]. Elevated ceramide levels have been found in the serum of patients with T2DM. Ceramide can inhibit tissue uptake of glucose by blocking AKT/PKB activation and inhibiting GLUT4 transport, which in turn leads to IR [35]. Related research shows that the level of HIF-1α expression is closely related to ceramide synthesis. For example, specific knockdown of HIF-1α in adipocytes can ameliorate the pathological process of atherosclerosis by inhibiting the production of ceramides [37]. Therefore, we speculate that Pue may inhibit ceramide production through the HIF-1α pathway, thus improving the pathological process of diabetes and providing a novel direction for studying how Pue functions in diabetes treatment. Our experiments similarly revealed that ceramide levels were significantly elevated in the liver of diabetic mice and that Pue reduced liver ceramide levels in diabetic mice.

It has been well-documented that ceramide synthases are closely related to IR and T2DM. Among them, SPTLC2 and SPTLC3 are the key rate-limiting enzymes essential for the initial step of ceramide synthesis from scratch. It has been reported in the literature that ceramide production was reduced when SPTLC2 was knocked out specifically in the liver of mice, thereby effectively protecting them from high-fat diet-induced obesity [38]. Similarly, the knockdown of SPTLC3 in HepG2 cells reduced ceramide levels and improved insulin sensitivity [39]. CERS2 and CERS6 are ceramide synthases involved in the ceramide de novo synthesis pathway. A study found that hepatocyte-specific knockdown of CERS2 inhibited the p38 MAPK and ERK1/ERK2 signaling pathways in mouse liver, thereby protecting mice from high-fat diet-induced hepatic steatosis and IR [40]. Liver-specific knockdown of CERS6 reduced C16:0 ceramide levels and protected mice from high-fat diet-induced obesity and glucose intolerance. In our study, RT-qPCR results showed that the expression of *SPTLC2* and *CERS2* mRNA increased in the livers of diabetic mice. In contrast, Pue reduced the expression of these genes in diabetic mice. This suggests that Pue may exert its therapeutic effect on diabetes by inhibiting the ceramide de novo synthesis pathway, which in turn reduces the level of ceramide in liver tissue.

In the current study, while we identified an interaction between Pue and HIF-1α using DARTS and molecular docking, the findings are preliminary and require further validation. Notably, the absence of evidence from advanced biophysical techniques such as Surface Plasmon Resonance (SPR) and MicroScale Thermophoresis (MST) is a significant limitation.

## 4. Materials and Methods

### 4.1. Reagents and Instruments

Puerarin (S30646, Shanghai Yuanye, Shanghai, China), palmitic acid (PA) (P0500, Sigma, St. Louis, MO, USA), bovine serum albumin V (BSA-V) (A8020, Solarbio, Beijing, China), STZ (S6089, Macklin, Shanghai, China), M-PER Mammalian Protein Extraction Reagent (78503, Thermo Fisher Science, Waltham, MA, USA), acetonitrile (A955-4, Thermo Fisher Science), methanol (A117-50, Thermo Fisher Science), formic acid (A456-4, Thermo Fisher Science), glucose assay kit (S0201M, Beyotime Biotechnology, Shanghai, China), ALT, AST, TG, TC, HDL-C, and LDL-C assay kits (C009-2-1, C010-2-1, A110-1-1,A111-1-1, A112-1-1, A113-1-1, Nanjing Jiancheng Bioengineering Institute, Nanjing, China), ceramide assay kit (M1037499, Shanghai Enzyme-linked Biotechnology, Shanghai, China), 96-well plates (BX-3599, Corning, Corning, NY, USA), 6-well plates (BX-3516, Corning, Corning, NY, USA), high-speed refrigerated centrifuge (D-37520, Sigma Laborzentrifugen GmbH, Osterode am Harz, Germany), microplate reader (Spark, Tecan Trading AG, Männedorf, Switzerland), blood glucose meter (cobas^®^ pulse, Roche, Basel, Switzerland).

### 4.2. Data Preparation for Network Pharmacology

First, gene expression data related to T2DM (dataset GSE23343) [41] and liver injury (dataset GSE54255) [42] were obtained from the GEO database. The GSE23343 dataset includes liver tissue samples from ten T2DM patients and seven individuals with normal glucose tolerance, while the GSE54255 dataset contains liver tissue samples from five liver injury patients and five healthy controls. Next, the gene expression matrices from both datasets were extracted, and differential expression analysis was performed using the “limma” package. The criteria for identifying DEGs were set as *p*-value < 0.05 and |log2FC| > 1. Subsequently, the “VennDiagram” package was employed to generate a Venn diagram of the DEGs from GSE23343 and GSE54255, identifying the shared DEGs between the two datasets. Furthermore, the “clusterProfiler” package was utilized to conduct gene ontology (GO) and KEGG pathway enrichment analyses on the shared DEGs to uncover their potential biological functions and involvement in signaling pathways. The shared DEGs were then imported into the STRING database to explore their potential protein–protein interaction (PPI) networks. The PPI network was then constructed, visualized, and analyzed using Cytoscape 3.10.1. Finally, the CytoHubba plugin was employed to apply nine different algorithms for screening hub genes within the PPI network, identifying hub genes for further functional correlation analysis.

### 4.3. Molecular Docking

In brief, referencing previous studies for molecular docking [43], the 2D structure of Pue (ID: 5281807) was obtained from the PubChem database. Next, the 3D configurations of HIF-1α (ID: 4H6J) and CXCL2 (ID: 5OB5) were extracted from the RCSbPDB database and prepared for upcoming molecular docking. PYMOL 2.5.5 software removed water and small molecule ligands from the target proteins. AutoDock 1.5.6 Tools software [44] was then used to hydrogenate the target protein, establish a molecular docking pocket, and finally perform molecular docking and visual analysis.

### 4.4. Cell Culture and Viability Analysis

HepG2 cells, obtained from the Cell Bank of the Chinese Academy of Sciences, were maintained in DMEM supplemented with 10% fetal bovine serum (FBS), 100 μg/mL streptomycin, and 100 U/mL penicillin. The cells were incubated at 37 °C with 5% CO_2_.

For the cell viability analysis, HepG2 cells were plated in 96-well plates and treated with varying concentrations of Pue (100, 50, 10, 5, 1, and 0.1 μM) for 24 h. After the treatment, CCK-8 reagent was added to each well and incubated for an additional 2 h. The optical density was then measured at 450 nm to assess cell viability [45].

### 4.5. Preparation of Palmitic Acid (PA) and Establishment of Insulin-Resistant HepG2 Cell Model

PA is a saturated fatty acid that can induce IR in hepatocytes. PA was prepared based on previous reports with slight modifications [46]. In brief, the appropriate amount of PA in sodium chloride solution was incubated at 70 °C for 30 min. Then, the PA was mixed with 20% BSA at a 1:1 ratio and filter-sterilized to obtain the stocked solution liquid of 5 mM PA-10% BSA.

When the confluence of HepG2 cells in the well plate reached 70–80%, the stock solution of 5 mM PA-10% BSA was diluted to the concentration (0.25 mM) using DMEM medium. The dilution was used to replace the existing culture medium for 24 h to establish the experimental model [47].

### 4.6. Detection of Glucose Intake

HepG2 cells were seeded in 96-well plates and cultured for 24 h. Then, the IR-HepG2 cell model was established according to Section 4.5. Meanwhile, cells were intervened with different concentrations of Pue (100, 50, 25 μM) or DMOG (500 μM) for 24 h according to set groupings. After centrifugation at 12,000× *g* for 5 min, the culture supernatant was taken and assayed according to the kit instructions, and the glucose intake of each group of cells was calculated. This experiment is slightly modified on the basis of previous reports [48].

### 4.7. Drug Affinity Responsive Target Stability

HepG2 cells were used to perform the DARTS experiment [31]. In short, HepG2 cells were treated with 0.25 mM PA-BSA-V for 24 h. After treatment, a mammalian protein extraction reagent was added, followed by centrifugation at 12,000 rpm for 10 min. The supernatant was retained, and 10× TNC buffer was added for protein quantification through the application of the BCA method. A working solution of 3 μg/μL protein lysate was prepared. The protein sample was then separated into three groups, each containing 199 μL. One group had 1 μL of DMSO added, while the other two groups had 1 μL of 20 mM or 100 mM Pue added before a 6 h incubation on ice. Next, we added proper protease or 1× TNC buffer for enzymatic hydrolysis. To terminate the reaction, an equivalent protease inhibitor was added, followed by a 10 min incubation on ice. Lastly, the 5× loading buffer was added and incubated for 10 min in a metal bath at 70 °C. Protein separation was carried out on sodium dodecyl sulfate–polyacrylamide gel electrophoresis (SDS-PAGE). The gels were stained with Coomassie brilliant blue at room temperature (RT) for 30 min.

### 4.8. Immunofluorescence

HepG2 cells were inoculated in 12-well plates containing slides, modeled, and administered in set groups. Subsequently, the plates were fixed with 4% paraformaldehyde for 30 min and then permeabilized with 0.2% Trition-100 for 10 min and then closed with 5% BSA at RT for 1 h. Primary antibodies were added for overnight incubation at 4 °C. At the end of incubation, a fluorescent secondary antibody was added and incubated at RT, protected from light for 1 h. Finally, the cell crawls were covered on slides containing an anti-fluorescence quenching sealer (containing DAPI) and observed using a fluorescence-inverted microscope.

### 4.9. Experimental Grouping and Drug Administration

Five-week-old male C57BL/6J mice (20 ± 2 g) were acquired from the Guangdong Medical Laboratory Animal Center. These mice were provided with food and water ad libitum under standard environmental conditions, which included a 12 h light and 12 h dark cycle, a controlled temperature of 24 ± 2 °C, and humidity levels of 50 ± 5%.

After adaptive feeding, eight mice were randomly selected as the control group. The remaining mice were fed with a high-fat diet for 7 weeks, followed by intraperitoneal injection of 45 mg/kg STZ solution for 5 consecutive days. One week after modeling, all mice were fasted with free access to water for 12 h. Afterward, the fasting blood glucose of the mice was detected by a blood glucose meter. When the blood glucose level reached 11.1 mmol/L, the model was considered successful [49]. Mice were then randomly divided into groups: model group (n = 8), positive drug treatment group (metformin 300 mg/kg, n = 8), Pue high-dose treatment group (P-H,160 mg/kg, n = 8), Pue low-dose treatment group (P-L, 80 mg/kg, n = 8), DMOG treatment group (8 mg/kg, n = 8), and DMOG + P-H treatment group (n = 8). After 13 weeks of intragastric administration, all mice were euthanized by isoflurane anesthesia.

The experimental procedures received approval from the Experimental Animal Ethics Branch of the Biomedical Ethics Committee of Guangdong Pharmaceutical University (registration number: gdpulacspf2017735-2). The mice were housed and fed at the Animal Center of Guangdong Pharmaceutical University.

### 4.10. Fasting Blood Glucose and Oral Glucose Tolerance Test

Blood was taken from the tail vein of mice by needle puncture, and fasting blood glucose was detected by a glucometer at 0, 4, 8, and 13 weeks of administration [50]. An oral glucose tolerance test was conducted in the 12th week of administration. After fasting for 12 h, mice in each group were orally administered 2 g·kg^−1^ of glucose, and then the blood glucose levels were measured at 0, 30, 60, 90, and 120 min post-administration using a blood glucose meter. The areas under the curve (AUC) of blood glucose levels were calculated to evaluate glucose tolerance.

### 4.11. Biochemical Analyses

After the mice were completely anesthetized, blood samples were collected from the heart. Subsequently, the mouse plasma was obtained by centrifugation at 3000 rpm for 10 min at 4 °C. Commercial assay kits were employed to quantitatively determine the plasma concentrations of AST, ALT, TC, TG, HDL-C, and LDL-C according to their respective instructions. The liver tissues were treated with PBS (1:10, *m*/*v*), homogenized, and centrifuged at 12,000 rpm for 20 min at 4 °C. The resulting supernatant was employed for ceramide detection using ELISA assay kits. After processing according to the corresponding assay kit, the data of all the above biochemical indicators were obtained using a microplate reader.

### 4.12. Histopathologic Examinations

Liver samples from diabetic mice were fixed in a 4% paraformaldehyde solution. These samples were sectioned after embedding in paraffin. Then, the liver tissue sections (4–7 μm) were stained with hematoxylin and eosin (H&E) to visualize cellular morphology. Additionally, liver samples were rapidly frozen (−20 °C), embedded in an optimal cutting temperature (OCT) compound, and sectioned into 6–8 μm frozen sections which were stained with oil red O to assess lipid accumulation. All histological sections were examined using an OLYMPUS SLIDEVIEW VS200 microscope (Tokyo, Japan) to obtain images for analysis.

### 4.13. Metabolomics Analysis

The liver sample (100 mg) was added to 350 μL of pre-chilled 70% methanol, homogenized, and centrifuged at 12,000 rpm for 20 min at 4 °C. This process was repeated twice, and the resulting supernatants were combined. The combined supernatant was then dried under a nitrogen stream and the residue was reconstituted in 100 μL of pre-cooled 50% methanol. After centrifuging for 20 min at 4 °C at 12,000 rpm, the supernatant was aliquoted into a sample vial for UPLC-Q-Exactive-MS analysis. The detailed methods are provided in the Supplementary Materials.

This experiment is slightly modified on the basis of previous reports [51,52]. In brief, the original data obtained were preprocessed with Compound Discoverer 3.1 software to obtain the molecular formula, mass-to-charge ratio, retention time, and peak area of compounds. The preprocessed data were imported into SIMCA 14.1 software for multivariate statistical analysis, and then the differential metabolites were screened using VIP > 1, *p* < 0.05. Finally, pathway enrichment analysis of differential metabolites was performed with MetaboAnalysis 5.0.

### 4.14. Real-Time Quantitative PCR (RT-qPCR)

The manufacturer’s protocol involved extracting total RNA from liver tissue using the RNA Easy Fast Tissue/Cell Kit. The extracted RNA was then reverse-transcribed into cDNA using the Evo M-MLV RT Mix Kit. Subsequently, RT-qPCR was performed in triplicate using the FastKing One-Step RT-PCR Kit in a Bio-rad CFX fluorescence quantitative PCR system (Bio-rad, Hercules, CA, USA). The results were normalized using β-actin and analyzed using the 2^ΔΔCT^ method. The primer sequences can be found in Supplementary Table S1.

### 4.15. Western Blot Analysis

The liver tissues were lysed in RIPA buffer with protease and phosphatase inhibitors, and the protein concentrations were measured using protein assay kits. A consistent quantity of 40 mg of protein was subjected to electrophoresis on an 8% SDS-PAGE. After electrophoresis, the proteins were blotted onto PVDF membranes. To minimize non-specific interactions, the membranes were preincubated with 5% skim milk at RT before being incubated with primary antibodies, including HIF-1α (20960-1-AP, Proteintech, Wuhan, Hubei, China), SPTLC2 (51012-2-AP, Proteintech, Wuhan, Hubei, China), and β-actin (66009-1-Ig, Proteintech, Wuhan, Hubei, China) at 4 °C overnight. After three washes with Tris Buffered Saline with Tween (TBST), the membranes were treated with HRP-linked secondary antibodies for 1.5 h at RT. Bands were detected using an ECL luminescent reagent kit, and their intensity was measured using ImageJ 1.53 analytical software.

### 4.16. Statistical Analysis

Data were analyzed by using GraphPad Prism 9.5 and SPSS 27.0 software. Data are presented as mean ± SEM. Statistical significance was determined using one-way ANOVA followed by Tukey’s post hoc test. *p* < 0.05 was considered statistically significant.

## 5. Conclusions

In conclusion, our comprehensive approach integrating bioinformatics, molecular docking, and metabolomics has elucidated the therapeutic potential of Pue in treating diabetic hepatopathy. Our findings identify HIF-1α as a central hub gene that links T2DM and liver injury, with its robust binding interaction with Pue positioning HIF-1α as a critical target for the therapeutic actions of Pue. Experimental evidence from both in vitro and in vivo studies indicates that Pue effectively improves IR and exhibits hepatoprotective properties. However, these beneficial effects are significantly counteracted by the HIF-1α stabilizer, DMOG. Furthermore, our metabolomic analysis reveals that Pue substantially modulates key metabolic pathways, particularly sphingolipid metabolism, which is intricately associated with the pathophysiology of diabetes and liver injury. The inhibition of ceramide production through the HIF-1α/SPTLC2 signaling pathway by Pue provides novel mechanistic insights into its therapeutic effects.

## Data Availability

Data is contained within the article or Supplementary Materials.

## Supplementary Materials

The following supporting information can be downloaded at: https://www.mdpi.com/article/10.3390/ph18030398/s1, Figure S1: DEGs and Pathway Enrichment Analysis in Liver Injury and T2DM; Table S1: Primer sequences for RT-qPCR analysis; Table S2: Gradient elution procedures; Table S3: Identification Information of Differential Metabolites (positive ion mode); Table S4: Identification Information of Differential Metabolites (negative ion mode).

## Abbreviations

The following abbreviations are used in this manuscript:

ALT	Alanine aminotransferase
AST	Aspartate aminotransferase
AUC	Area under curve
BSA	Bovine serum albumin
CERS2	Ceramide synthase 2
DARTS	Drug affinity responsive target stability
DEGs	Differentially Expressed Genes
DMOG	Dimethyloxallyl glycine
FBG	Fasting blood glucose
GO	Gene Ontology
H&E	Histopathologic examinations
HDL-C	High-density lipoprotein cholesterol
HFD	High-fat diet
HIF-1α	Hypoxia inducible factor-1α
IR	Insulin resistance
KEGG	Kyoto Encyclopedia of Genes and Genomes
LDL-C	Low-density lipoprotein cholesterol
OGTT	Oral glucose tolerance test
PA	Palmitic acid
PPI	Protein-Protein Interaction Networks
Pue	Puerarin
PVDF	Polyvinylidene fluoride
RT	Room temperature
ROS	reactive oxygen species
SPTLC2	Serine palmitoyltransferase 2
STZ	Streptozotocin
T2DM	Type 2 diabetes mellitus
TC	Total cholesterol
TG	Triglyceride
UPLC	Ultra-high performance liquid chromatography
