# Supplementary material for: Puerarin Targets HIF-1α to Modulate Hypoxia-Related Sphingolipid Metabolism in Diabetic Hepatopathy via the SPTLC2/Ceramide Pathway
Source: Pharmaceuticals (Basel). 2025 Mar 12;18(3):398. doi: 10.3390/ph18030398 (PMC11945571; doi:10.3390/ph18030398)
Supplement: Supplementary file 1 [file pharmaceuticals-18-00398-s001.zip › pharmaceuticals-3483520-supplementary.pdf]

Table S1 Primer sequences for RT-qPCR analysis.

Gene	Sequences
β-actin-F	GTACCACCATGTACCCAGGC
β-actin-R	AACGCAGCTCAGTAACAGTCC
PHD2-F	AGCATACGCCACAAGGTACG
PHD2-R	TACTTTAGCTCTCGCTCGCTC
PHD3-F	CAGACCGCAGGAATCCACAT
PHD3-R	TTCAGCATCGAAGTACCAGACAGT
SPTLC2-F	AACGGGGAAGTGAGGAACG
SPTLC2-R	CAGCATGGGTGTTTCTTCAAAAG
CERS2-F	ATGCTCCAGACCTTGTATGACT
CERS2-R	CTGAGGCTTTGGCATAGACAC

UPLC-Q/TOF-MS analysis

Chromatographic conditions

Chromatographic column: Waters ACQUITY UPLC ® BEH C18 column (2.1 mm × 100 mm × 1.7 µM); Column temperature: 30 ℃; Flow rate: 0.3 mL/min; Sample plate temperature: 4 ℃; Mobile phase: 0.1% formic acid-H2O(A) and 0.1% formic acid-acetonitrile(B); Sample volume: 5 µL; Gradient elution procedures are shown in Table S2.

Table S2 Gradient elution procedures

**Time(min)**	**A (%)**	**B (%)**
0	90	10
5	90	10
15	50	50
25	10	90
30	10	90
35	90	10
38	90	10

Mass Spectrometry Conditions

Electrospray ionization (ESI) in positive and negative ion modes; Sheath gas flow rate: 30 arb; Auxiliary gas pressure: 10 arb; Dryer flow rate: 10 L/min; Spray voltage: 3.5 KV; Ion source temperature: 350 ℃; Auxiliary gas temperature: 200 ℃; Scanning mode: FULL MS; Scanning range: 100~1600 m/z; Collision energy: 30, 40, 50 ev.

Table S3 Identification Information of Differential Metabolites (positive ion mode)

**Proposed Identity**	**Formula**	**M/Z**	**HMDB/KEGG ID**
Cinnamic acid	C9H8O2	148.05226	HMDB0000567
Hypoxanthin	C5H4N4O	136.03829	HMDB0000157
L-(+)-Leucine	C6H13NO2	131.09439	HMDB0000687
DL-Phenylalanine	C9H11NO2	165.07869	HMDB0000062
1-Linoleoyl-2-Hydroxy-sn-glycero-3-PC	C26H50NO7P	519.33215	HMDB0010386
Maleamic acid	C4H5NO3	115.02675	METPA0190
3-Methylsulfolene	C5H8O2S	132.02434	HMDB0059667
Reduced Glutathione	C10H17N3O6S	307.08337	HMDB0000125
1-Hexadecanoylpyrrolidine	C20H39NO	309.30276	HMDB0032740
L-Proline	C5H9NO2	115.06323	HMDB0000162
1-(4Z,7Z,10Z,13Z,16Z,19Z-docosahexaenoyl)-sn-glycero-3-phosphocholine	C30H50NO7P	567.33213	HMDB0010404
DL-Glutamine	C5H10N2O3	146.06894	HMDB0000641
L-Pyroglutamic acid	C5H7NO3	129.04241	HMDB0000267

1-Nitrosopiperidine	C5H10N2O	114.07926	HMDB0243970
(E)-p-coumaric acid	C9H8O3	164.04712	HMDB0002035
Rimexolone	C24H34O3	370.25021	HMDB0015033
Amide C18	C18H37NO	283.28695	HMDB0034146
L-(-)-methionine	C5H11NO2S	149.05092	HMDB0000696
L-(-)-Serine	C3H7NO3	105.04237	HMDB0000187
Itaconic acid	C5H6O4	130.0264	HMDB0002092
quinbolone	C24H32O2	352.23964	HMDB0242660
7-ketodeoxycholic acid	C24H38O5	406.2712	HMDB0000391
ZV4	C5H11NO	101.08393	HMDB0256275
7alpha-Hydroxy-3-oxochol-4-en-24-oic acid	C24H36O4	388.26066	HMDB0062744
Creatine	C4H9N3O2	131.0693	HMDB0000064
4-Aminobenzoic acid	C7H7NO2	137.04753	HMDB0001392
2,2'-Iminodipropan-1-ol	C6H15NO2	133.11005	HMDB0251354
1-arachidonoyl-sn-glycero-3-phosphocholine	C28H50NO7P	543.33174	HMDB0010395
Taurine	C2H7NO3S	125.01448	HMDB0000251
DL-Histidine	C6H9N3O2	155.06928	HMDB0003412

Phenylisocyanate	C7H5NO	119.03692	HMDB0062270
Xanthine	C5H4N4O2	152.03315	HMDB0000292
235BBF3K97	C20H28	268.21859	HMDB0062447
1-[(8Z,11Z,14Z)-icosatrienoyl]-sn-glycero-3-phosphocholine	C28H52NO7P	545.3473	HMDB0010393
L-(+)-ERGOTHIONEINE	C9H15N3O2S	229.08825	HMDB0003045
Acetylcarnitine	C9H17NO4	203.11556	HMDB0000201
L-(-)-Threonine	C4H9NO3	119.05811	HMDB0000167
Coumarone	C8H6O	118.04168	HMDB0032929
O-propenoyl-D-carnitine	C10H17NO4	215.11552	HMDB0013124
uridine 5′-diphosphate	C9H14N2O12P2	404.00164	HMDB0000295
MFCD00025555	C9H18N2O3	202.13184	HMDB0028691
lysophosphatidylethanolamine (22:6(4Z,7Z,10Z,13Z,16Z,19Z)/0:0)	C27H44NO7P	525.28502	HMDB0011526
MFCD00133435	C26H52NO7P	521.34776	HMDB0002815
C14-Carnitine	C21H41NO4	371.3031	HMDB0005066
3-Methylglutarylcarnitine	C13H23NO6	289.15218	HMDB0000552

N(1)-acetylspermidine	C9H21N3O	187.16825	HMDB0001276
AC 45594	C13H20O2	208.14593	HMDB0246434
UDP-GlcNAc	C17H27N3O17P2	607.0808	HMDB0000290
1-[(9Z)-hexadecenoyl]-sn-glycero-3-phosphocholine	C24H48NO7P	493.31612	HMDB0010383
N-{(2S,3R,4E)-3-Hydroxy-1-[(3-O-sulfo-beta-D-threo-hexopyranosyl)oxy]-4-octadecen-2-yl}octadecanamide	C42H81NO11S	807.55492	HMDB0012314
[(2R,3S,4R,5R)-5-(6-Amino-9H-purin-9-yl)-3,4-dihydroxytetrahydro-2-furanyl]methyl [(2R,3S,4R,5R)-3,4,5-trihydroxytetrahydro-2-furanyl]methyl dihydrogen diphosphate	C15H23N5O14P2	559.07153	HMDB0303049
MFCD00010474	C27H44O2	400.33353	HMDB0000501
(9R,23Z)-1-[(2R,3S,4R,5R)-5-(6-Amino-9H-purin-9-yl)-4-hydroxy-3-(phosphonooxy)tetrahydro-2-furanyl]-3,5,9-trihydroxy-8,8-dimethyl-10,14,19,21-tetraoxo-2,4,6-trioxa-18-thia-11,15-diaza-3,5-	C29H44N7O20P3S	935.15581	HMDB0304148

diphosphahex acos-23-en-26-oic acid 3,5-dioxide			
Timonacic	C4H7NO2S	133.01962	HMDB0258979
Tris(hydroxymethyl)aminomethane	C4H11NO3	121.07371	HMDB0240288
(11R,23R)-14,17,20-Trihydroxy-23-(octanoyloxy)-14,20-dioxido-8,26-dioxo-9,13,15,19,21,25-hexaoxa-14lambda~5~,20lambda~5~-diphosphatritriacontan-11-yl decanoate	C43H82O17P2	932.50448	HMDB0116798
(-)-Prostaglandin E2	C20H32O5	352.22448	HMDB0001220
[3,3'-{8-[(4E,8E)-1-Hydroxy-5,9,13-trimethyl-4,8,12-tetradecatrien-1-yl]-3,7,12,17-tetramethyl-13-vinyl-2,18-porphyrindiyl-kappa~2~N~22~,N~24~}dipropanoato(2-)]iron	C49H58FeN4O5	838.37358	HMDB0001162
MFCD00135810	C11H17NO8	291.09512	HMDB0341246
cis-5-Tetradecenoylcarnitine	C21H39NO4	369.28746	HMDB0002014
S-{1-[(2R,3S,4R,5R)-5-(6-Amino-9H-purin-9-yl)-4-hydroxy-3-(phosphonooxy)tetrahydro-2-furanyl]-	C30H49N8O18P3S	934.20802	HMDB0006403

3,5,9-trihydroxy-8,8-dimethyl-3,5-dioxido-10,14-dioxo-2,4,6-trioxa-11,15-diaza-3lambda~5~,5lambda~5~-diphosphaheptadecan-17-yl} (2S,3S)-3-hydroxy-8-methyl-8-azabicyclo[3.2.1]octane-2-carbothioate			
1-(1H-Imidazol-5-yl)-3-(phosphonooxy)-2-propanone	C6H9N2O5P	220.02549	HMDB0012236
(3S)-3-Isopropenyl-6-oxoheptanoyl-CoA	C31H50N7O18P3S	933.21019	METPA1022
1,3-distearoyl-2-oleoylglycerol	C57H108O6	888.81517	HMDB0005368
N-[(2S)-2-Hydroxypropanoyl]methionine	C8H15NO4S	221.07179	HMDB0062182
Retinyl acetate	C22H32O2	328.24013	HMDB0035185
2,3-Dihydroxyolean-12-ene-23,28-dioic acid	C30H46O6	502.32884	HMDB0034551
D-Tryptophyl-D-alanyl-D-allothreonylglycyl-D-histidyl-L-phenylalanyl-D-methioninamide	C40H53N11O8S	847.37861	HMDB0013017
9,12-Hexadecadienoylcarnitine	C23H41NO4	395.30316	HMDB0013334
4-{(Z)-[(5E,8Z,11Z,14Z)-1-Hydroxy-5,8,11,14-icosatetraen-1-ylidene]amino}butanoic acid	C24H39NO3	389.29185	HMDB0062330

lactide	C6H8O4	144.04203	HMDB0246077
propionylcarnitine	C10H19NO4	217.13242	HMDB0000824
D-Glucosamine 6-phosphate	C6H14NO8P	259.04556	HMDB0001254
Cefozopran	C19H17N9O5S2	515.08116	HMDB0249772
PC(14:1(9Z)/16:1(9Z))	C38H72NO8P	701.50133	HMDB0007903
N-Palmitoyltyrosine	C25H41NO4	419.30242	HMDB0062340
paspaline	C28H39NO2	421.29738	C20530
3-Palmito-1,2-Diolein	C55H102O6	858.76659	HMDB0005382
L-(+)-Valine	C5H11NO2	117.07877	HMDB0250806
(6E,10E)-3,7,11,15-Tetramethyl-6,10,14-hexadecatrien-1-ol	C20H36O	292.27648	HMDB0032147
Indoleacrylic acid	C11H9NO2	187.06303	HMDB0000734
MFCD00036904	C24H50NO7P	495.33156	HMDB0010382
(7Z,19R)-25-Amino-22-hydroxy-22-oxido-16-oxo-17,21,23-trioxa-22lambda~5~-phosphapentacos-7-en-19-yl (4Z,7Z,10Z,13Z,16Z,19Z)-4,7,10,13,16,19-docosahexaenoate	C43H72NO8P	761.49846	HMDB0008979

1-oleoyl-2-linoleoyl-sn-glycero-3-phosphocholine	C44H82NO8P	783.57772	HMDB0008105
ditekiren	C50H75N9O8	929.56975	HMDB0251488
2,6-di-tert-butylhydroquinone	C14H22O2	222.16169	HMDB0040178
Linoleyl carnitine	C25H45NO4	423.33427	HMDB0006469
MFCD22416941	C25H47NO4	425.34978	HMDB0005065
Propamocarb	C9H20N2O2	188.15229	HMDB0341191
Phenprocoumon	C18H16O3	280.10948	HMDB0015081
(9Z)-9-Octadecenamide	C18H35NO	281.27141	HMDB0247607、HMDB0002117
C14-Dihydroceramide	C32H65NO3	511.49559	HMDB0011759
C16-Dihydroceramide	C34H69NO3	539.52661	HMDB0011760
5-Carboxy-8-hydroxy-2-(4-hydroxy-3,5-dimethoxyphenyl)pyrano[4,3,2-de]chromen-1-ium-3-yl 6-O-[(2E)-3-(4-hydroxyphenyl)-2-propenoyl]-D-glucopyranoside	C35H31O16	707.16081	HMDB0029239
gamma-Aminobutyric acid	C4H9NO2	103.06294	HMDB0000112
N-octodecanoylsphinganine	C36H73NO3	567.55802	HMDB0011761

Table S4 Identification Information of Differential Metabolites (negative ion mode)

Proposed Identity	Formula	M/Z	HMDB/KEGG ID
taurohyocholic acid	C26H45NO7S	515.29081	HMDB0011637
1-O-(9,16,17-Trihydroxy-18-oxokauran-18-yl)hexopyranose	C26H42O10	514.27809	HMDB0035096
Uridine diphosphate glucose	C15H24N2O17P2	566.05395	HMDB0000286
UDP-GlcNAc	C17H27N3O17P2	607.08083	HMDB0000290
Glucoheptonic Acid	C7H14O8	226.06796	HMDB0014471
[(2R,3S,4R,5R)-5-(6-Amino-9H-purin-9-yl)-3,4-dihydroxytetrahydro-2-furanyl]methyl [(2R,3S,4R,5R)-3,4,5-trihydroxytetrahydro-2-furanyl]methyl dihydrogen diphosphate	C15H23N5O14P2	559.07062	HMDB0303049
beta-D-Ethyl glucuronide	C8H14O7	222.07298	HMDB0010325
6-O-Phosphonohex-2-ulofuranose	C6H13O9P	260.02912	HMDB0000124
Erythrose, 4-phosphate	C4H9O7P	200.00797	HMDB0001321
N-Acetyl-L-glutamic acid	C7H11NO5	189.06279	HMDB0001138
Arabic acid	C5H10O6	166.04674	HMDB0000539
Adenylsuccinic acid	C14H18N5O11P	463.07317	HMDB0000536

D-Sucrose	C12H22O11	342.1155	HMDB0000258
threonic acid	C4H8O5	136.03711	HMDB0000943
ribulose 5-phosphate	C5H11O8P	230.0184	HMDB0000618
phosphopantothenic acid	C9H18NO8P	299.07625	HMDB0062700
Flavin mononucleotide	C17H21N4O9P	456.1039	HMDB0001520
Plumbagin	C11H8O3	188.04822	HMDB0035291
Retinyl acetate	C22H32O2	328.23977	HMDB0035185
leucodelphinidin	C15H14O8	322.07003	METPA1713
S-(Formylmethyl)glutathione	C12H19N3O7S	349.09355	HMDB0060507
Inosinic acid	C10H13N4O8P	348.04639	HMDB0000175
cholic acid	C24H40O5	408.28682	HMDB0000619
Linoelaidic Acid	C18H32O2	280.23988	HMDB0006270
7-ketodeoxycholic acid	C24H38O5	406.27104	HMDB0000391
D-PANTOTHENIC ACID	C9H17NO5	219.10987	HMDB0000210
N-Gluconyl ethanolamine phosphate	C8H18NO10P	319.06642	HMDB0032294
Indoxyl sulfate	C8H7NO4S	213.00875	HMDB0000682
(+/-)-2-Hydroxyglutaric acid	C5H8O5	148.03687	HMDB0002323

(4S)-4-[(6-Carboxyhexanoyl)oxy]-4-(trimethylammonio)butanoate	C14H25NO6	303.16767	HMDB0013328
(E)-p-coumaric acid	C9H8O3	164.04626	HMDB0002035
ecabet	C20H28O5S	380.16505	HMDB0015613
loviride	C17H16Cl2N2O2	350.06001	HMDB0254178
Chlorogenic acid	C16H18O9	354.09401	HMDB0003164
Dexelvucitabine	C9H10FN3O3	227.07131	HMDB0251085
(3aR,4R,5R,6aS)-5-Hydroxy-4-[(1E,3S)-3-hydroxy-1-octen-1-yl]hexahydro-2H-cyclopenta[b]furan-2-one	C15H24O4	268.16711	HMDB0341299
Ethyl malate	C8H14O5	190.08323	HMDB0040220
6-O-Phosphono-D-gluconic acid	C6H13O10P	276.02379	HMDB0001316
(1E,5Z)-14-[1-(1H-Indol-3-yl)ethyl]-4,6,15,15a-tetramethyl-9,10,14,14a,15,15a,16a,16b-octahydro-3H-cyclotrideca[d]oxireno[f]isoindole-7,8,11,12(4H,13H)-tetrone	C33H38N2O5	542.27601	HMDB0031919
1-(sn-glycero-3-phospho)-1D-myo-inositol	C9H19O11P	334.06593	HMDB0011649

Norepinephrine sulfate	C8H11NO6S	249.02989	HMDB0002062
N,O-DIDESMETHYLVENLAFAXINE-GLUCURONIDE	C21H31NO8	425.20418	HMDB0061167
4-[7-(beta-D-Glucopyranosyloxy)-4-oxo-4H-chromen-3-yl]phenyl beta-D-glucopyranosiduronic acid	C27H28O15	592.14478	HMDB0041719
Phosphoenolpyruvic acid	C3H5O6P	167.9814	HMDB0000263

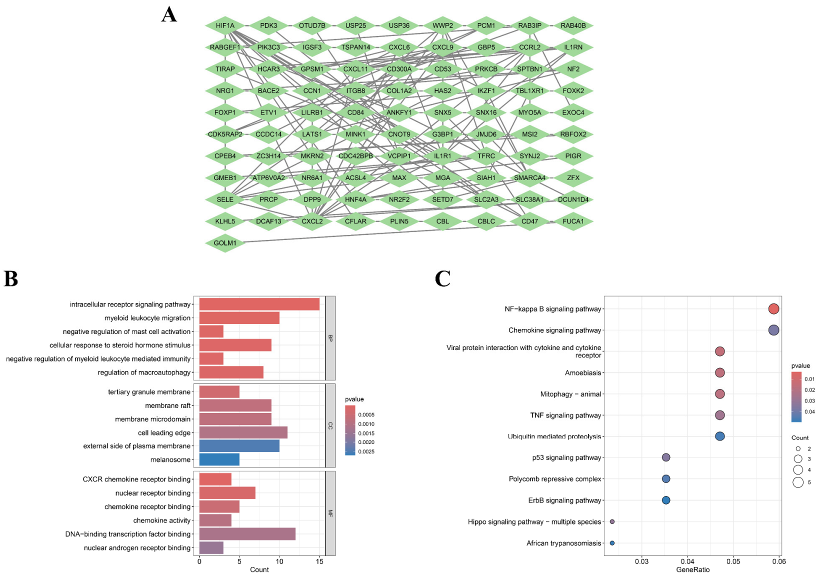

Figure S1. DEGs and Pathway Enrichment Analysis in Liver Injury and T2DM. (A) PPI network; (B). GO analysis; (C) KEGG analysis.
